# Supplementary material for: Quantitative Analysis of Polysaccharide Composition in Polyporus umbellatus by HPLC–ESI–TOF–MS
Source: Molecules. 2019 Jul 10;24(14):2526. doi: 10.3390/molecules24142526 (PMC6681038; doi:10.3390/molecules24142526)
Supplement: Supplementary file 1 [file molecules-24-02526-s001.pdf]

Supporting information

Quantitative analysis of polysaccharide composition in *Polyporus umbellatus* by HPLC-ESI-TOF-MS

Ning Guo [1], Zongli Bai [2], Zongtao Lin [3], Weijuan Jia [1], Jianhua Sun [2], WanWan Wang [1], JingJing Dong [2], Shizhong Chen [1,*] and Hong Wang [1,*]

[1] School of Pharmaceutical Sciences, Peking University, Beijing 100191, China; Guoning19900906@163.come (N.G.); Jiaweijiuan517@163.com (W.J.); 1450373611@qq.com (W.W.)

[2] Kangmei Pharmaceutical Co.Ltd, Puning 515300, China; 3050471@qq.com (Z.B.); lyhwkj@126.com (J.S.); 18811331279@163.com (J.D.)

[3] Department of Pharmaceutical Sciences, University of Tennessee Health Science Center, Memphis, TN 38163, United States; zongtao@sas.upenn.edu (Z.L.)

* Correspondence: hw9505@bjmu.edu.cn (H.W.); chenshizhong66@163.com (S. C.)

Table S1. Factors and levels of the orthogonal $L_9(3)^4$ experiments.

	Factor	level		
		**1**	**2**	**3**
A	Hydrolysis time (h)	3	6	9
B	hydrolysis temperature (ºC)	100	110	120
C	concentration of TFA (M)	2	3	4

Table S2. The orthogonal experiment results of glucose released from PPS.

No.	A (h)	B (ºC)	C (M)	D[2]	Peak area (mAu × min)
1	1	1	1	1	19832553
2	1	2	2	2	19418943
3	1	3	3	3	23857274
4	2	1	2	3	21008328
5	2	2	3	1	25607274
6	2	3	1	2	26377491
7	3	1	3	2	23530119
8	3	2	1	3	24444606
9	3	3	2	1	23069984
K_1	21036257	21457000	23551550	22836604	
K_2	24331031	23156941	21165752	23108851	
K_3	23681570	24434916	24331556	23103403	
R[1]	3294774.3	2977916.3	3165804	272247.33	

1 R refers to the result of extreme analysis

2 D refers to the error column used to the variance analysis.

Table S3. The orthogonal experiment results of glucosamine released from PPS.

No.	A (h)	B (ºC)	C (M)	D[2]	Peak area (mAu × min)
1	1	1	1	1	310528
2	1	2	2	2	238940
3	1	3	3	3	162996
4	2	1	2	3	305686
5	2	2	3	1	192207
6	2	3	1	2	169919
7	3	1	3	2	283933
8	3	2	1	3	169849
9	3	3	2	1	176813
K_1	237488	300049	216765.33	226516	
K_2	222604	200332	240479.67	230930.67	
K_3	210198.3	169909.33	213045.33	212843.67	
R[1]	27289.67	130139.67	27434.33	18087	

1 R refers to the result of extreme analysis

2 D refers to the error column used to the variance analysis.

Table S4. The orthogonal experiment results of glucuronic acid released from PPS.

No.	A (h)	B (ºC)	C (M)	D[2]	Peak area (mAu × min)
1	1	1	1	1	640585
2	1	2	2	2	548991
3	1	3	3	3	1108463
4	2	1	2	3	708610
5	2	2	3	1	1225162
6	2	3	1	2	1235617
7	3	1	3	2	831719
8	3	2	1	3	1177904
9	3	3	2	1	688376
K_1	766013	726971.33	1018035.33	851374.33	
K_2	1056463	984019	648659	872109	
K_3	899333	1010818.67	1055114.67	998325.67	
R[1]	290450	283847.33	406455.67	146951.33	

1 R refers to the result of extreme analysis

2 D refers to the error column used to the variance analysis.

Table S5. Variance analysis results of glucose released from PPS.

Variation sources	Quadratic sum	Degree of freedom	Mean square	F[4]	P[5]
A[1]	1.8275E+13	2	9.1375E+12	125.748	< 0.05
B[2]	1.3391E+13	2	6.6955E+12	92.142	< 0.05
C[3]	1.6323E+13	2	8.1614E+12	112.315	< 0.05
D (Error)	1.4533E+11	2	7.2665E+10	1	

$F_{0.05}(2, 2) = 19.00$

[1] A is the hydrolysis temperature.

[2] B is the sulfuric acid concentration.

[3] C is the hydrolysis time.

[4] F is the critical value.

[5] P is the probability value.

Table S6. Variance analysis results of glucosamine released from PPS.

**Variation sources**	**Quadratic sum**	**Degree of freedom**	**Mean square**	**F**[4]	**P**[5]
A[1]	1.1202E+9	2	5.6008E+8	2.099	>0.05
B[2]	2.7805E+10	2	1.3902E+10	52.113	< 0.05
C[3]	1.3289E+9	2	6.6443E+8	2.491	> 0.05
D (Error)	5.3356E+8	2	2.6678E+8	1	

$F_{0.05}$ (2, 2) =19.00

[1] A is the hydrolysis temperature.

[2] B is the sulfuric acid concentration.

[3] C is the hydrolysis time.

[4] F is the critical value.

[5] P is the probability value.

Table S7. Variance analysis results of glucuronic acid released from PPS.

**Variation sources**	**Quadratic sum**	**Degree of freedom**	**Mean square**	**F[4]**	**P[5]**
A[1]	1.2683E+11	2	6.3413E+10	3.341	>0.05
B[2]	1.4736E+11	2	7.3681E+10	3.382	> 0.05
C[3]	3.0302E+11	2	1.5151E+11	7.984	> 0.05
D (Error)	3.7955E+10	2	1.8978E+10	1	

$F_{0.05}$ (2, 2) =19.00

[1] A is the hydrolysis temperature.

[2] B is the sulfuric acid concentration.

[3] C is the hydrolysis time.

[4] F is the critical value.

[5] P is the probability value.
